# Amperometric Biosensors Based on Direct Electron Transfer Enzymes

**DOI:** 10.3390/molecules26154525

**Published:** 2021-07-27

**Authors:** Franziska Schachinger, Hucheng Chang, Stefan Scheiblbrandner, Roland Ludwig

**Affiliations:** Biocatalysis and Biosensing Laboratory, Department of Food Sciences and Technology, BOKU–University of Natural Resources and Life Sciences, 1190 Vienna, Austria; franziska.schachinger@boku.ac.at (F.S.); hucheng.chang@boku.ac.at (H.C.); stefan.scheiblbrandner@boku.ac.at (S.S.)

**Keywords:** biosensor, current density, direct electron transfer, enzyme, selectivity, sensitivity, nanomaterial, self-assembled monolayers, nanocomposite, nanoparticle

## Abstract

The accurate determination of analyte concentrations with selective, fast, and robust methods is the key for process control, product analysis, environmental compliance, and medical applications. Enzyme-based biosensors meet these requirements to a high degree and can be operated with simple, cost efficient, and easy to use devices. This review focuses on enzymes capable of direct electron transfer (DET) to electrodes and also the electrode materials which can enable or enhance the DET type bioelectrocatalysis. It presents amperometric biosensors for the quantification of important medical, technical, and environmental analytes and it carves out the requirements for enzymes and electrode materials in DET-based third generation biosensors. This review critically surveys enzymes and biosensors for which DET has been reported. Single- or multi-cofactor enzymes featuring copper centers, hemes, FAD, FMN, or PQQ as prosthetic groups as well as fusion enzymes are presented. Nanomaterials, nanostructured electrodes, chemical surface modifications, and protein immobilization strategies are reviewed for their ability to support direct electrochemistry of enzymes. The combination of both biosensor elements—enzymes and electrodes—is evaluated by comparison of substrate specificity, current density, sensitivity, and the range of detection.

## 1. Introduction

### 1.1. Background

Chemical sensors consist of a chemical recognition system, a physicochemical transducer and a signal processing unit that transforms the concentration of the analyte into a quantifiable signal. A biosensor employs a biological recognition (biorecognition) element working on the principle of bioaffinity or biocatalysis. Bioaffinity sensors use receptor proteins, antibodies, or polynucleotides as biorecognition elements, whereas enzymes are used as bioelectrocatalysts. Several types of signal transducers can be distinguished, for example: mass-sensitive, optical, and electrochemical. Electrochemical biosensors can be based on the detection of a measurable current (amperometric), the accumulation of charge (potentiometric), changes in current as a result of a potentiometric effect at a gate electrode (field effect), changes in the conductive properties of a medium between electrodes (conductometric), or the changes of resistance and reactance close to an electrode (impedimetric).

### 1.2. Amperometric Biosensors

**Figure 1:**
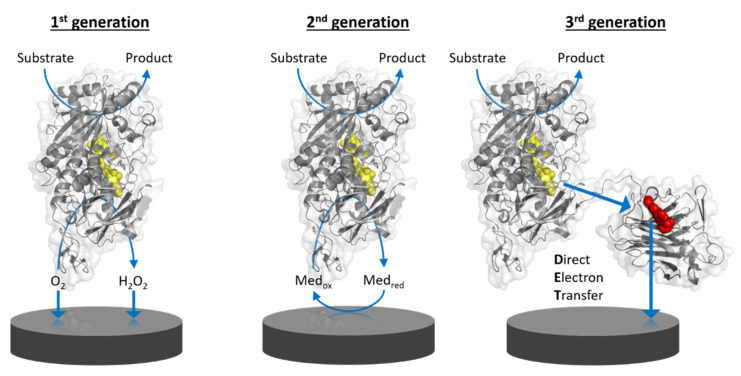
Schematic representation of the working principle of the three generations of enzyme-based amperometric biosensors. 1st generation: based on the electrocatalytic detection of enzymatic substrate conversion or product formation. 2nd generation: based on the electrocatalytic recycling of a redox mediator. 3rd generation: based on direct electron transfer from enzyme to electrode. Yellow molecules depict the FAD cofactor of flavoenzymes and the red molecule depicts the heme cofactor in the mobile cytochrome domain of a multi-cofactor enzyme.

Amperometric biosensors utilizing oxidoreductases were classified into three generations (Figure 1): 1st generation biosensors employing oxidases based on the electrocatalytic monitoring of substrate consumption or product formation, 2nd generation biosensors employing oxidases or dehydrogenases based on the electrocatalytic recycling of suitable redox mediators, and 3rd generation biosensors employing preferentially dehydrogenases capable of direct electron transfer to bare or modified electrodes. Direct electron transfer (DET) forms the basis of 3rd generation biosensors [1]. DET is the ability of a solubilized or immobilized redox active protein to exchange electrons with an electrode upon contact; for example, oxidoreductases directly donating electrons gained from substrate oxidation to the electrode. Therefore, 3rd generation biosensors can be operated at lower potentials (close to the midpoint potential of the enzyme’s prosthetic group) than those needed for the detection of enzymatic reaction products, e.g., H_2_O_2_, in 1st generation biosensors, or even the redox potential needed to apply redox mediators to shuttle electrons between the enzyme cofactor and the electrode in 2nd generation biosensors. The specificity of 3rd generation biosensors is thus less affected by electroactive interferents like ascorbic acid and also by the absence of the redox active mediator.

### 1.3. Applications for DET-Based Biosensors

**Figure 2:**
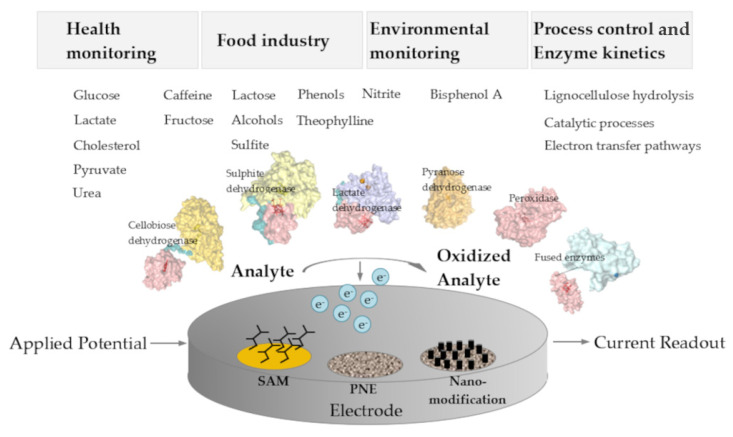
Schematic overview on application areas, analytes, enzymes, and the architecture of 3rd generation amperometric biosensors. SAM, self-assembled monolayer; PNE, porous nanostructured electrodes.

Potential applications of amperometric biosensors range from the monitoring of environmental markers like pesticides, medical analytes in blood or urine like glucose, lactate, and cholesterol, biomarkers for cancer or diabetes, to various industrially important substances as saccharides, alcohols, and phenols for process monitoring. Moreover, biosensors for the monitoring of cellulose-degrading enzymes and lignocellulosic biomass hydrolysis have been proposed [2,3]. Interestingly, enzymes for application in 3rd generation amperometric biosensors are mostly heme, flavo-, or quino-enzymes, but copper enzymes are also known to interact with electrodes through DET (Figure 2).

### 1.4. Challenges of DET-Based Biosensors

The prerequisite of direct electrochemistry with a reasonably high DET rate is a close proximity of the enzyme’s prosthetic group to the electrode surface, since the electron transfer rate decreases exponentially with the distance, i.e., by a factor of ~10^4^ when the distance is increased from 8 to 17 Å [4,5,6,7]. However, many articles claim DET, e.g. for glucose oxidase employed in glucose biosensors, but omit the presentation of appropriate data and reference experiments. DET is likely to occur when (1) the onset potential of the electrocatalytic reaction is close to the redox potential of the prosthetic group in the enzyme, (2) a significant catalytic current is measured upon substrate addition but does not occur when adding a similar substance that is not a substrate of the enzyme (e.g. L-glucose), and (3) the experimental design rules out the presence of free cofactors which can serve as redox mediators or other electroactive substances generating current. Notably, electrocatalytic currents obtained from the oxidation of H_2_O_2_ (H_2_O_2_→2H^+^ + O_2_ + 2e^−^, E^0^ = 0.69 V vs. NHE [8]), or weakly bound coenzymes or prosthetic groups like NAD^+^/NADH [9,10,11] or PQQ [12], may feign DET but actually act as redox mediators. The close distance between the cofactor and the electrode surface necessary for DET can be achieved either by employing single cofactor, multi-cofactor, or fusion enzymes with surface-exposed prosthetic groups or by means of electrode design and engineering employing chemical or nanomaterial modifications like carbon nanotubes, graphene, and their nanocomposites, which serve as electron relay between the buried redox center and the electrode [13] or the linking of artificial redox mediators to the enzyme itself [14,15,16].

### 1.5. Scope

Direct electron transfer of biological recognition elements to electrodes crucially depends on the nature and architecture of the employed enzyme on the one hand and on the surface properties and structures of the electrode, on the other hand. This review covers enzymes, for which DET has been shown and which have been used as biorecognition elements in biosensors: oxidases, dehydrogenases, peroxidases, P450 cytochromes, or copper oxidases with single prosthetic groups such as FAD, FMN, heme, PQQ, molybdenum, iron sulfur clusters or copper (Section 2.1), but also enzymes with more than one prosthetic group (multi-cofactor enzymes, Section 2.2) or genetically engineered fusion enzymes (Section 2.3). Additionally, electrode designs and engineering that facilitate or enhance the DET properties of employed enzymes are discussed.

For an overview on electrochemical sensors and biosensors, their working principle and classification, as well as potential applications, the work of Kimmel et al. is recommended [17]. A deeper focus on biosensors only, providing key achievements, reasons for successful applications, emerging technologies, and information on the future role of biosensors in healthcare and wellbeing, is given by Turner [18,19]. A comprehensive survey on electrochemical biosensors providing details on the different types of biosensing techniques is given by Ronkainen et al. [20] and Grieshaber et al. [21].

## 2. Enzymes

### 2.1. Single Cofactor Enzymes

Single cofactor enzymes exhibiting DET can contain FAD, FMN, PQQ, or heme as prosthetic group. The DET rate of single cofactor enzymes is limited by the accessibility of the cofactor which is part of an active site typically deeply buried within the enzyme. A random electron transfer due to exposed redox centers certainly is a disadvantage to the natural role of most enzymes. Flavin cofactors have a lower redox potential and are typically deeper buried compared to PQQ prosthetic groups, that sometimes are even surface exposed. PQQ however has higher redox potentials, which make it less prone to random electron transfer processes. The electron transfer pathways of most enzymes are not well studied. 

#### 2.1.1. Peroxidases

**Figure 3:**
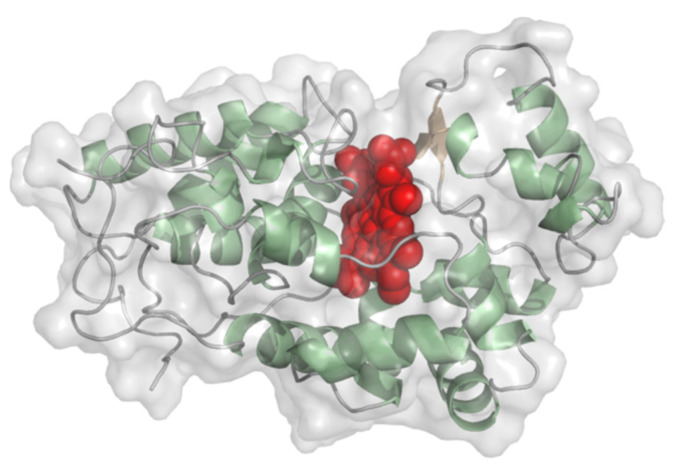
Crystal structure of *Armoracia rusticana* peroxidase (PDB: 1ATJ) with its heme cofactor (red spheres) almost protruding from the protein surface.

Horseradish peroxidase (EC 1.11.1.7, peroxidase) and other peroxidases convert various phenolic substrates with H_2_O_2_ as co-substrate and produce phenoxy radicals and H_2_O. The molecular weight of horseradish peroxidase is around 35 kDa and the overall structure is built mostly from helices. A short two-stranded beta-sheet is found in close vicinity to the heme prosthetic group (Figure 3). The rather exposed heme is ligated to one histidine. The second ligand position is occupied by the substrate. The redox potential of horseradish peroxidase was determined to be in the range of −300 to −270 mV vs. NHE at pH 7 [22,23] and −310 mV vs. NHE at pH 7 for soybean peroxidase [24]. DET was demonstrated for horseradish, tobacco, peanut, sweet potato [25], and soybean peroxidase [26]. Because of the consumption of H_2_O_2_, peroxidases are used for various bi-enzymatic sensors, e.g., for glucose monitoring together with glucose oxidase, methyl salicylate with alcohol dehydrogenase/horseradish peroxidase combination, but also for H_2_O_2_ producing copper enzymes like uricase or tyrosinase [27,28,29,30,31]. For such bi-enzyme sensors that have peroxidase co-immobilized with the analytical enzyme, it has to be considered that mediated electron transfer by electrochemical peroxide oxidation on the electrode is also possible and that the enzyme-mediated conversion of H_2_O_2_ by the peroxidase resulting in DET signal occurs simultaneously as was also discussed by Lindgren et al. [25]. The group searched for peroxidases with higher catalytic activity, which are suitable for the design of reliable biosensors and suggested sweet potato peroxidase as a suitable candidate.

A recombinant horseradish peroxidase-based 3rd generation biosensor employing a polycrystalline gold electrode for H_2_O_2_ detection showed a sensitivity of 1400 µA mM^−1^ cm^−2^ [32]. Another 3rd generation biosensor for peroxide detection based on soybean peroxidase—less prone to losing its heme than horseradish peroxidase—on glassy carbon electrodes coated with single-walled carbon nanohorns (cone-shaped graphene sheets) exhibited a sensitivity of 16.625 µA mM^−1^ [33]. Tobacco peroxidase has been evaluated for biosensors since it has several advantages over horseradish peroxidase such as stability in a broader pH range or higher stability towards inactivation by H_2_O_2_ [34,35]. Entrapment of the peroxidase on a thiol-modified gold electrode yielded multifold higher currents compared to horseradish peroxidase, which however might be the result of protein orientation on the self-assembled monolayer (SAM) [34]. Tobacco, horseradish, and peanut peroxidase were applied on SAM-modified gold electrodes and graphite electrodes and compared on their phenol, aniline, *o*- and *p*- aminophenol, and *o*-, *m*-, and *p*- phenylenediamine detection. Distinct substrate specificities and sensitivities were observed for each peroxidase [35].

#### 2.1.2. Theophylline Oxidase Cytochrome P450

Theophylline oxidase is a cytochrome P450 enzyme and contains heme as prosthetic group. Theophylline is a methyl-derivative of xanthine and is physically relevant for treatment of bronchial asthma and chronic obstructive pulmonary disease (COPD), but it is also a component in tea beverages. DET for the theophylline oxidase was demonstrated on gold and graphite electrodes and a midpoint potential of +100 mV vs. NHE was reported [36,37]. The electrochemical characterization of the enzyme in the presence of non-natural electron acceptors was done by Shipovskov and Ferapontova [38]. Direct and mediated electron transfer were studied on graphite or SAM-modified gold electrodes and by entrapment within an osmium-complexed polymer, respectively [39]. Catalytic currents were measured with cyclic voltammetry. The authors demonstrated that the DET strongly depends on the nature of the SAM. The sensitivities of the DET- and MET-based biosensors with a phospholipid-modified graphite electrode and a SAM-modified gold electrode were 0.05 and 0.01 µA mM^−1^ cm^−2^, respectively, and 52.1 µA mM^−1^ cm^−2^ with the osmium polymer-modified gold electrode in the physiologically relevant range of 3.6–72 mg mL^−1^.

#### 2.1.3. Glucose Oxidase

**Figure 4:**
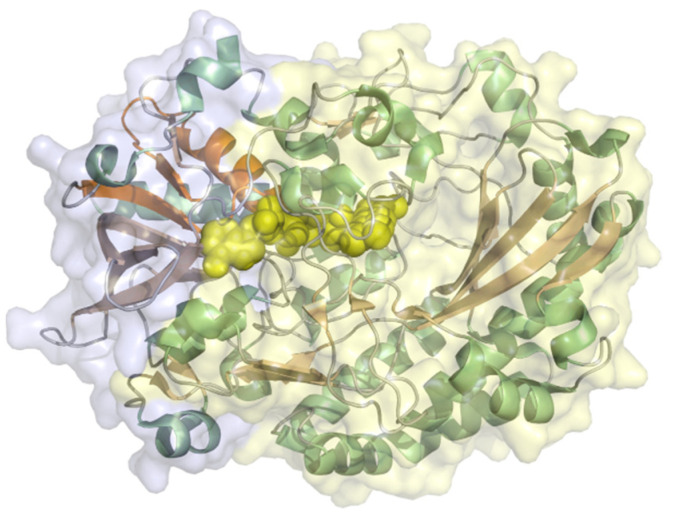
Crystal structure of *Aspergillus niger* glucose oxidase (PDB: 3QVR) with the FAD cofactor (yellow spheres) buried inside the protein. The FAD-binding and substrate-binding subunits are colored light blue and light yellow, respectively. The GMC-oxidoreductase family characteristic dinucleotide binding βαβ-motif (Rossmann-fold) is highlighted in orange.

FAD-dependent enzymes converting glucose have been intensively studied for blood glucose monitoring in diabetes management. Glucose oxidase (EC 1,1.3.4, β-D-glucose:oxygen 1-oxidoreductase) is a flavoenzyme catalyzing the oxygen dependent oxidation of β-D-glucose to D-glucono-1,5-lactone and H_2_O_2_. The 65 kDa, N-glycosylated protein with an FAD cofactor rather buried inside the protein shell is kidney-shaped and comprises an FAD-binding subunit featuring the dinucleotide binding βαβ-motif (Rossmann-fold) and a substrate binding subunit with a prominent six-stranded beta sheet forming the substrate pocket (Figure 4). It is usually applied in commercially available 1st generation glucose biosensors due to its high glucose specificity and high turnover numbers. Because of H_2_O_2_ production and a dependency on the surrounding O_2_ concentration, glucose oxidase is inherently unsuitable for 3rd generation biosensors. In continuously operated glucose monitoring devices, a 2nd generation design employing redox mediators or redox polymers can reduce the effects of O_2_ conversion. Further literature on the current state of general glucose sensor design can be found in [40]; this review gives an overview on the success and challenges in the development of glucose biosensors and also addresses problems associated with off-target detection of saccharides other than glucose.

There is a disagreement in the field whether glucose oxidase is capable of DET or not. Typically, DET is claimed for a combination of glucose oxidase with a nanomaterial. Bartlett and Al-Lolage, however, could not confirm DET of glucose oxidase but instead disproved some commonly claimed DET scenarios for glucose oxidase [41] by demonstrating that detected signals can originate from enzyme-dissociated FAD or from FAD impurities present in commercially obtained preparations, which can feign DET. They recommend keeping five considerations in mind before claiming DET: (1) the nature of the enzyme (and likelihood for DET), (2) the purity of the enzyme, (3) the electrocatalytic response and its magnitude upon substrate addition, (4) reference experiments with affine but not convertible substrates (i.e., L-glucose in case of glucose oxidase), and (5) reference experiments with convertible substrates that have other reaction kinetics. However, they list a small number of articles, where DET from glucose oxidase to modified electrodes seemed evident (see Section 3.3).

#### 2.1.4. FAD-Dependent Glucose Dehydrogenase

**Figure 5:**
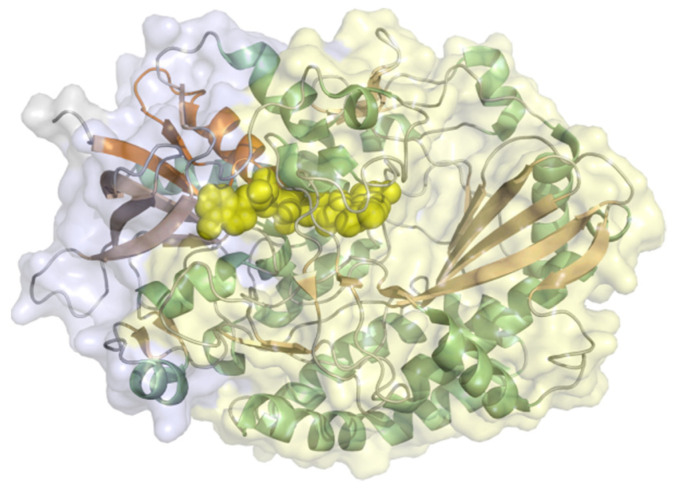
Crystal structure of *Aspergillus flavus* glucose dehydrogenase (PDB: 4YNT) with the FAD cofactor (yellow spheres) buried inside the protein shell. The FAD-binding and substrate-binding subunits are colored light blue and light yellow, respectively. The GMC-oxidoreductase family characteristic dinucleotide binding βαβ-motif (Rossmann-fold) is highlighted in orange.

Glucose dehydrogenase (EC 1.1.5.9, glucose 1-dehydrogenase) is a flavoenzyme occurring predominantly in fungi. Fungal glucose dehydrogenase has FAD as prosthetic group (Figure 5) and belongs to the family of GMC-oxidoreductases [42]. Therefore, we handle reports of fungal glucose dehydrogenase capable of DET with the same precautions as for glucose oxidase. An example of DET for the fungal *Aspergillus terreus* glucose dehydrogenase on porous gold modified with Pt-nanoclusters was reported with an extraordinarily high current of ~1 mA cm^−2^ recorded at 0 V vs. Ag|AgCl [43]. However, in control experiments, the authors also demonstrated that glucose can be oxidized directly by the electrode and the addition of FAD reduced the catalytic current which does not point towards an enzyme catalyzed reaction. Bacterial glucose dehydrogenases are structurally distinct enzymes also carrying FAD, but with additional multiheme subunits (see Section 2.2.2).

#### 2.1.5. FAD-Domain of Cellobiose Dehydrogenase

**Figure 6:**
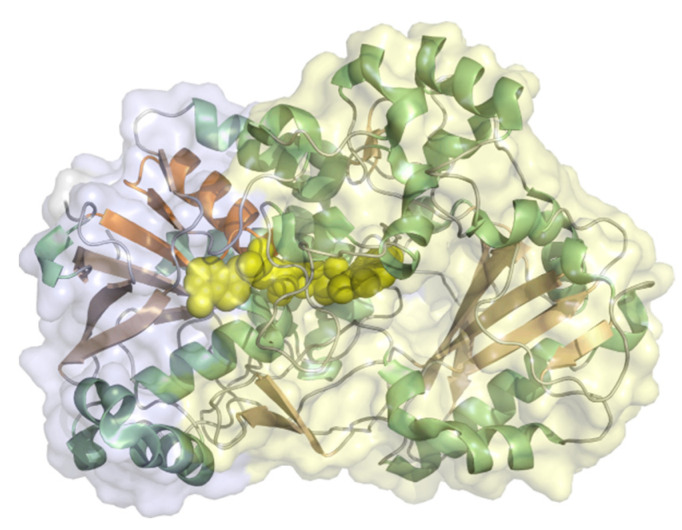
Crystal structure of the dehydrogenase domain of *Neurospora crassa* cellobiose dehydrogenase (PDB: 4QI7) with the FAD cofactor (yellow spheres) buried inside the protein shell. The FAD-binding and substrate-binding subunits are colored light blue and light yellow, respectively. The GMC-oxidoreductase family characteristic dinucleotide binding βαβ-motif (Rossmann-fold) is highlighted in orange.

The FAD-dependent dehydrogenase domain of cellobiose dehydrogenase (EC 1.1.99.18, cellobiose dehydrogenase acceptor, Figure 6)—the full-length protein is discussed later in Section 2.2.1—converts cellobiose to cellobiono-1,5-lactone but can convert other saccharides such as lactose, too. Concerning DET, the same precautions as for glucose oxidase and glucose dehydrogenase apply. Schulz et al. demonstrated DET for the dehydrogenase domain of cellobiose dehydrogenases below pH 5.0 with mercaptohexanol- and mercaptoundecanol-modified gold electrodes and the enzyme entrapped on the electrode under a dialysis membrane [44]. Catalytic currents were demonstrated with lactose as substrate clearly with midpoint potentials lower than that of the full-length protein where DET originates from the cytochrome domain. The authors also distinguished between currents originating from free or bound FAD. Kielb et al. immobilized a cellobiose dehydrogenase on PDADMAC-coated metal electrodes and showed through a combination of electrochemical and surface-enhanced resonance Raman spectroscopic measurements a Ca^2+^-induced orientation of the enzyme on the electrode surface such that a DET pathway from the FAD to the electrode can be formed [45]. However, all obtained currents were low and of limited use for amperometric biosensors.

#### 2.1.6. FAD-Dependent Pyranose Dehydrogenase

**Figure 7:**
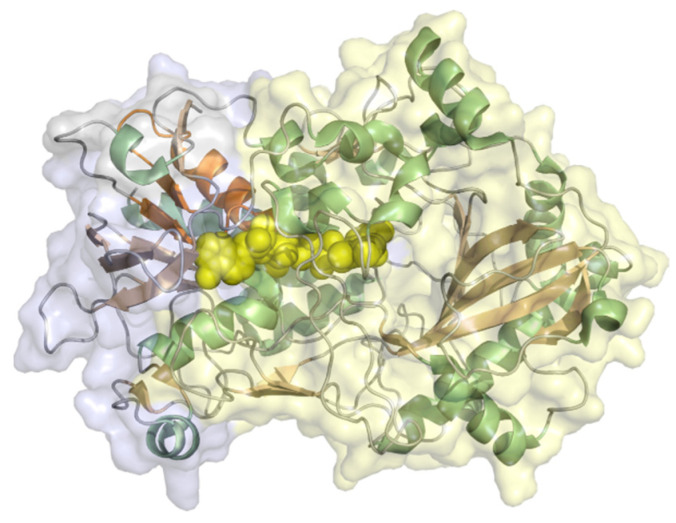
Crystal structure of *Agaricus meleagris* pyranose dehydrogenase (PDB: 4H7U) with the FAD cofactor (yellow spheres) buried inside the protein shell. The FAD-binding- and substrate-binding subunits are colored light blue and light yellow, respectively. The GMC-oxidoreductase family characteristic dinucleotide binding βαβ-motif (Rossmann-fold) is highlighted in orange.

Fungal FAD-pyranose dehydrogenase (EC 1.1.99.29, Figure 7) of *Agaricus meleagris* showed DET on graphite, but only when the protein was fully deglycosylated [46]. A glycosylation shell is necessary for long term protein stability, which is in turn desirable for the application in 3rd generation biosensors [47]. Cyclic voltammetry was performed in this publication, oxidative and reductive peaks were not observed at low concentration of the protein, but catalytic currents and peaks were observed with increasing protein concentration. The midpoint potential of the enzyme is around +150 mV vs. NHE, which is consistent with spectrocelectrochemically determined values of the covalently bound prosthetic group. 

#### 2.1.7. PQQ-Dependent Glucose Dehydrogenase

For enzymes with a pyrroloquinoline quinone (PQQ) prosthetic group, DET is challenging to prove. The low affinity of the prosthetic group results in a significant concentration of PQQ in solution that can function as redox mediator. Thus, PQQ-dehydrogenases might be a good option for single-use measurement devices but are probably not suitable for continuous long-term applications. PQQ-dependent enzymes applied in biosensors can be single cofactor enzymes like PQQ-glucose dehydrogenase or multi-cofactor enzymes like PQQ-pyranose dehydrogenase featuring a cytochrome *b* domain or PQQ-alcohol dehydrogenase which are discussed later in this review.

**Figure 8:**
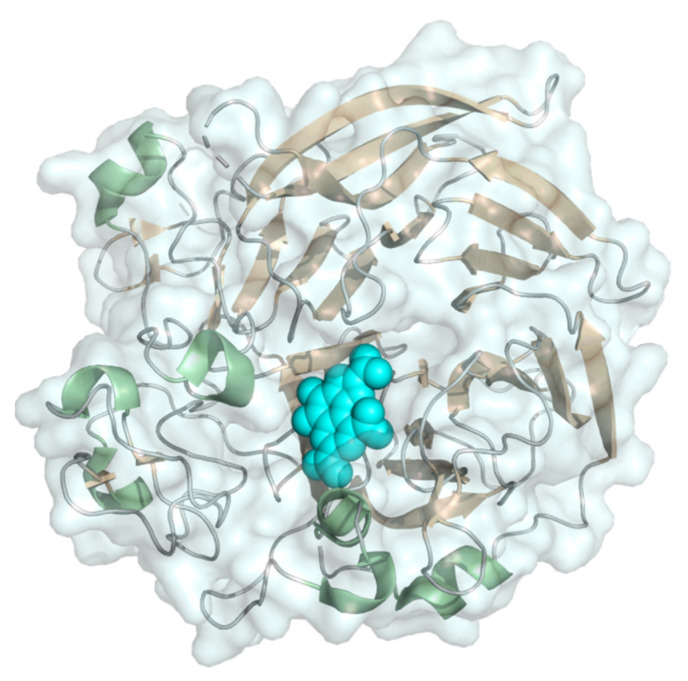
Crystal structure of *Acinetobacter calcoaceticus* PQQ-dependent glucose dehydrogenase (PDB: 5MIN) with the PQQ cofactor (cyan spheres) rather exposed to the protein surface.

PQQ-glucose dehydrogenase was found to exist as soluble and as membrane-bound protein with very low sequence identity. Soluble PQQ glucose dehydrogenase (EC 1.1.99.17 glucose dehydrogenase (pyrroloquinoline-quinone) is a homodimer. The size of the monomer is ~50 kDa and the structure (Figure 8) comprises six repeats of a four-stranded anti-parallel beta-sheet, all together forming a beta-propeller fold [48]. Calcium ions were found to stabilize the dimeric complex but also the cofactor [48]. The membrane-integrated PQQ glucose dehydrogenase (EC 1.1.5.2, glucose 1-dehydrogenase PQQ, quinone) from *Acinetobacter* is also a homodimer and does use calcium and magnesium to stabilize its structure [49]. Its catalytic cycle is not affected by O_2_, which is a major advantage. The DET of the soluble-type PQQ-glucose dehydrogenase was reported for a carbon cryogel with currents of 0.93 mA cm^−2^ at +400 mV vs. NHE [50] or on MWCNT-modified gold electrodes with 0.5 mA cm^−2^ at +300 mV vs. NHE [51]. Milton and Minteer, however, state that PQQ-dependent glucose dehydrogenase is not capable of DET [52].

#### 2.1.8. PQQ-Domain of Pyranose Dehydrogenase

**Figure 9:**
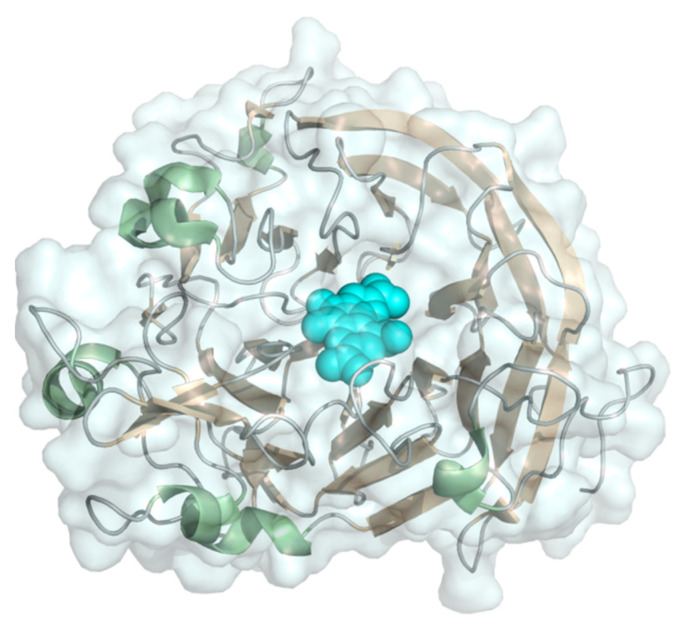
Crystal structure of the dehydrogenase domain of *Coprinopsis cinerea* PQQ-dependent pyranose dehydrogenase (PDB: 6JWF) with the PQQ cofactor rather exposed to the protein surface.

The recently discovered eukaryotic PQQ-dependent pyranose dehydrogenase (EC 1.1.2.B5, PQQ-dependent quinohemoprotein pyranose dehydrogenase, Figure 9) comprises a substrate oxidizing pyrroloquinoline-quinone dependent subunit and a cytochrome domain homologous to cellobiose dehydrogenase [53]. Its PQQ-domain was produced and tested for DET when immobilized on Au-nanoparticle modified gold disc electrodes and used for the detection of L-fucose in urine samples as a cancer indicator. A sensitivity of 3.12 ± 0.05 μA mM^−1^ cm^−2^ and a detection limit of 13.6 μM was demonstrated [54,55]. As already discussed, it must be taken in consideration that the PQQ cofactor in this enzyme is also not tightly bound and might function as a diffusing redox mediator.

#### 2.1.9. Copper Oxidases

(Multi)copper enzymes for which DET was demonstrated are laccase [56,57,58], bilirubin oxidase [59] galactose oxidase [60], ascorbate oxidase [61], tyrosinase [62], and non-enzyme copper electron transfer proteins as ceruloplasmin [63], azurin [64], and hemocyanin [65]. (Multi)copper oxidases mostly have a high redox potential of >350 mV vs. NHE [66,67,68,69] and use O_2_ as co-substrate, which results in the formation of either H_2_O_2_ or H_2_O. Biosensors based on (multi)copper oxidases are usually based on the detection of O_2_ consumption or H_2_O_2_ formation which would interfere with the high potentials required to detect DET of (multi)copper enzymes. Therefore, it is not practicable to construct 3rd generation biosensors with these enzymes. However, due to their high redox potential the multicopper oxidases laccase and bilirubin oxidase are often employed as DET cathode bioelectrocatalysts in biofuel cells.

### 2.2. Multi-Cofactor Direct Electron Transfer Enzymes

Multi-cofactor enzymes carry more than one prosthetic group. Often multi-cofactor enzymes have an additional heme domain for guided electron transfer chains additionally to their catalytic groups. These cytochrome domains are either loosely bound and connected by a linker (i.e., cellobiose dehydrogenase, FMN-dependent lactate dehydrogenase, sulfite dehydrogenase, PQQ-dependent pyranose, or alcohol dehydrogenase) or tightly bound, structurally close domains (i.e., xanthine oxidase and bacterial multi-cofactor enzymes). Flavoprotein dehydrogenases usually interact with *b*-type cytochrome domains, PQQ can have interacting cytochromes with either a *b*-type or a *c*-type heme. Dehydrogenases presented here are either fungal extracellular enzymes or bacterial dehydrogenases. Cytochrome-linked dehydrogenases are ideal enzymes for 3rd generation biosensors due to their optimized electron transfer via well exposed cytochrome domains. 

#### 2.2.1. Cellobiose Dehydrogenase

**Figure 10:**
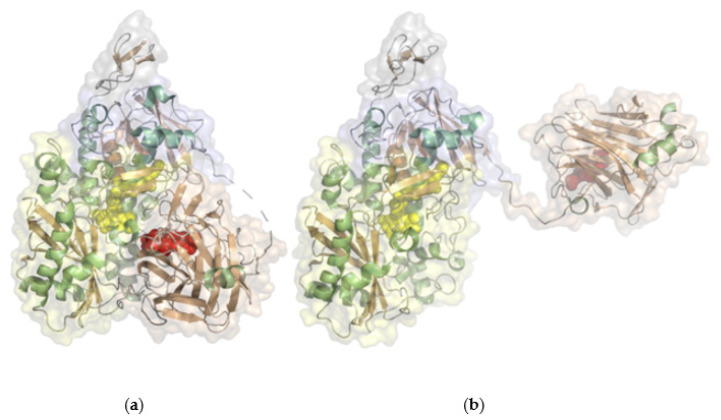
Crystal structure of (**a**) *Crassicarpon hotsonii* cellobiose dehydrogenase in closed conformation (PDB: 4QI6) and (**b**) *Neurospora crassa* cellobiose dehydrogenase in open conformation (PDB: 4QI7) with the FAD cofactor (yellow spheres) buried inside the protein shell. The FAD-binding- and substrate-binding subunits are colored light blue and light yellow, respectively. The GMC-oxidoreductase family characteristic dinucleotide binding βαβ-motif (Rossmann-fold) is highlighted in orange. A C-terminal type 1 carbohydrate binding module (CBM_1) is shown “on top” of the structure. The DET-competent cytochrome domain (light red) with the surface exposed heme b cofactor (red spheres) accepts electrons from substrate oxidation from the FAD in the closed conformation and donates them to suitable acceptors in the open conformation.

Cellobiose dehydrogenase (EC 1.1.99.18, cellobiose dehydrogenase (acceptor)) consists of a FAD-dependent dehydrogenase domain (DH) linked via a flexible linker to a cytochrome *b* domain (CYT) that is specifically found in such fused oxidoreductase-cytochrome enzymes (Figure 10). Some cellobiose dehydrogenases feature a C-terminal type 1 carbohydrate binding module (CBM_1). The molecular weight is typically 90 kDa with two-thirds attributed to the dehydrogenase domain, which is built from a FAD-binding subunit with the βαβ-dinucleotide binding motif (Rossmann-fold) and a substrate binding subunit. Recently, a class of CDHs without the typical cytochrome domain was also identified [42], but it contains few species. The flexible linker domain has conserved residues and is optimized to allow for domain movement to conduct interdomain electron transfer and DET to another protein or electrodes. The dehydrogenase domain is specific for cellobiose but can also convert lactose or glucose [70]. The DET of a cellobiose dehydrogenase was first reported in 1996 using a graphite electrode and later for thiol-modified gold electrodes [71,72]. Redox potentials originating from the heme *b* domain at pH 5 were reported from +100 to +180 mV vs. NHE [70,72]. Numerous cellobiose dehydrogenase-based 3rd generation biosensors have been reported, like lactose biosensors based on a spectroscopic graphite rod electrode [73]. A sensitivity of 9.8 µA mM^−1^ cm^−2^ with a linear detection range between 2 and 150 µM substrate was reported for the sensor based on *Trametes villosa* cellobiose dehydrogenase and a sensitivity of 11 µA mM^−1^ cm^−2^ with a linear range of 1–100 µM substrate for the *Phanerochaete sordida* cellobiose dehydrogenase-based electrode. Cellobiose dehydrogenase applications in biosensors and bioelectrochemical features of various cellobiose dehydrogenases were recently reviewed [70,74]. The first commercially available 3rd generation biosensor has been developed by DirectSens GmbH. The lactose specific biosensor LactoSensR is designed to detect very low lactose concentrations in lactose-reduced or lactose-free dairy products [75].

#### 2.2.2. Bacterial FAD-Dependent Glucose Dehydrogenase

Bacterial glucose dehydrogenases comprise a FAD-dependent catalytic (α-) subunit additionally harboring a surface-exposed iron/sulfur cluster [76], a multiheme electron transfer (β-) subunit, and a chaperone-like (γ-) subunit. The advantages of FAD carrying glucose dehydrogenases are the very low oxidase activity and their low turnover number of maltose. Yamashita and coworkers employed a bacterial glucose dehydrogenase from *Burkholderia cepacia* for sensors. This FAD-glucose dehydrogenase has a cytochrome subunit that enables DET [77]. The authors mention that this glucose dehydrogenase has a broader substrate specificity but their engineered variant is claimed to be less specific towards maltose. A nafion/Lionpaste W311N (an aqueous Ketjen Black dispersion) and glutaraldehyde vapor cross-linked enzyme mix were applied on disposable carbon screen-printed electrode chips. Measurements were performed at 250 mV vs. Ag|AgCl, where currents reached up to 6 µA. Gineityte and colleagues reported the DET of the *Ewingella americana* glucose dehydrogenase on gold nanoparticle and also achieved current densities up to 1 mA cm^−2^ [78]. Ratautas detected 10 mA cm^−2^ at −0.22 V vs. SCE with the same protein and also glucose as substrate on a 4-aminothiophenol-modified Au-nanoparticle electrode [79].

#### 2.2.3. Bacterial FAD-Dependent Fructose Dehydrogenase

Fructose dehydrogenase (EC 1.1.99. 11, fructose 5-dehydrogenase) from *Gluconobacter* sp. is a membrane-bound enzyme and consists of a FAD-dependent dehydrogenase subunit in complex with three covalently linked cytochrome *c* subunits. Its natural substrate is D-fructose. Direct electron transfer of fructose dehydrogenase has been demonstrated several times [80,81,82]. The influence of the shape of Au-nanotriangles and -nanospheres on graphite electrodes [83] and the pH and divalent/monovalent cations [84] on the DET properties of fructose dehydrogenase has been investigated. A highly sensitive 3rd generation fructose sensor based on polycrystalline gold electrodes electrodeposited with highly porous gold showed an extraordinary stability of 90 days, a linear range between 0.05 and 5 mM, and a sensitivity of 175 ± 15 μA mM^−1^ cm^−2^ [85].

#### 2.2.4. FMN-Dependent L-Lactate Dehydrogenase

**Figure 11:**
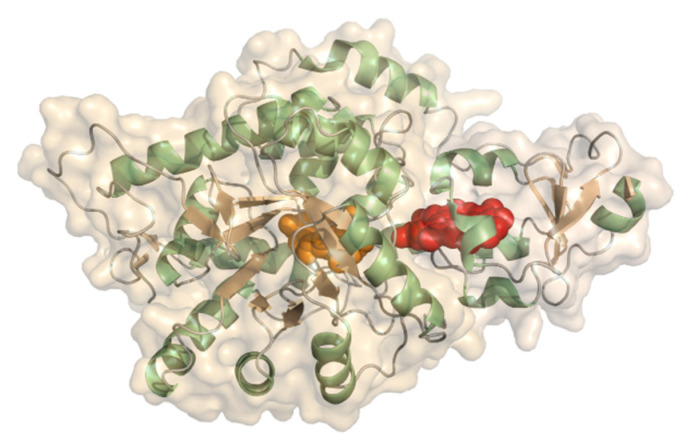
Crystal structure of *Saccharomyces cerevisiae* L-lactate dehydrogenase (PDB: 1KBI) with the FMN (orange spheres) and the heme *b* (red spheres) cofactors in close electron transfer-favorable vicinity to each other.

L-Lactate dehydrogenase or flavocytochrome *b*_2_ (EC 1.1.2.3, L-lactate dehydrogenase (cytochrome)) is a two-domain protein forming a homotetramer with an FMN (flavin mononucleotide) consisting of a dehydrogenase domain and a cytochrome *b* domain, linked by a short linker (Figure 11). It is ~57 kDa in size and the N-terminal heme domain is structurally a *b_5_*-type cytochrome. The dehydrogenase domain carries a buried FMN. L-lactate dehydrogenase has been studied from baker’s yeast (*Saccharomyces cerevisiae*) and *Hansenula polymorpha* (syn. *Ogataea angusta*). The cytochrome domain is structurally almost identical to typical eukaryotic *b*_5_-type cytochromes. The homotetrameric structure of the baker’s yeast enzyme was reported early on [86,87]. L-lactate dehydrogenase converts lactate to pyruvate. The redox potential was determined to be +8 mV by redox titration at pH 7 [88]. The midpoint potential of the isolated cytochrome was further determined on thiol-modified gold electrodes to be in the range of −50 to −30 mV vs. NHE from pH 5–9. L-lactate dehydrogenase was demonstrated to perform DET to carbon-based electrodes for lactate detection [89,90]. *Hansenula polymorpha* L-lactate dehydrogenase was originally studied on differently modified graphite electrodes using mediators [91]. Later, the same group further developed 3rd generation sensors using Au-nanoclusters with a sensitivity of 10.6 µA mM^−1^ cm^−2^ [92]. Lactate concentrations within human saliva and sweat samples were also quantified with this sensor.

#### 2.2.5. Xanthine Oxidase

**Figure 12:**
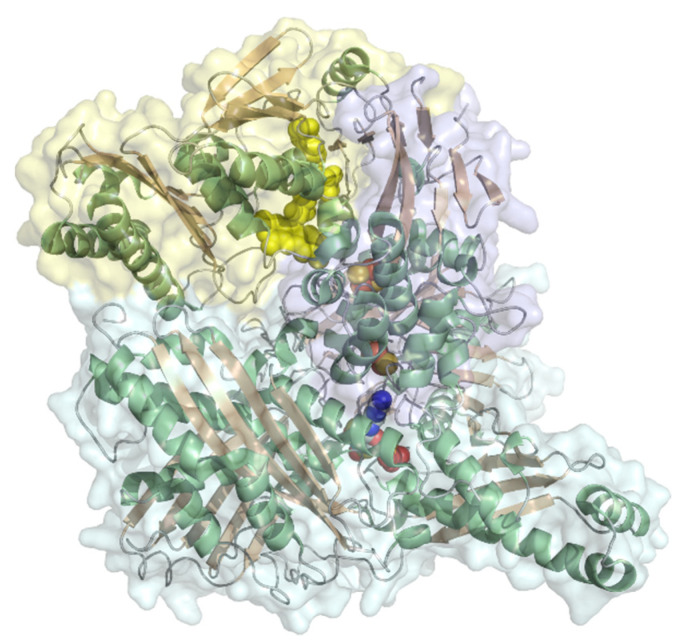
Crystal structure of *Bos taurus* xanthine oxidase (PDB: 1FIQ) comprising the iron/sulfur (light blue), the FAD (light yellow), and the molybdopterin (light cyan) domains. The respective cofactors are shown as colored spheres (FAD, yellow; iron/sulfur, red/orange; Mo-pterin, red/blue).

Xanthine oxidase (EC 1.17.3.2, hypoxanthine-xanthine oxidase) catalyzes a reaction from xanthine to urate with H_2_O_2_ (or under specific conditions superoxide) as a byproduct but can also oxidize other purines, pterins, and aldehydes. It is a heteromer with a 20 kDa iron-sulfur cluster subunit, a 40 kDa FAD subunit, and 84 kDa molybdopterin-binding subunit and a total molecular weight of almost 290 kDa for the heterodimeric structure (Figure 12). All subunits are mixed α/β structures with few flexible loops. Xanthine oxidoreductases in mammals are expressed as an NAD^+^-dependent dehydrogenase but are steadily converted to oxidases by oxidation of sulfhydryl residues, catalytic cleavage, or oxidation of cysteine thiols to disulfide bonds [93]. The conversion to the oxidase also occurs during purification. Xanthine oxidase was immobilized on colloidal laponite, a synthetic silicate-based clay-modified glassy carbon electrodes as a DET-based xanthine biosensor. The midpoint potential was found to be −126 mV vs. NHE and the sensitivity, limit of detection, and linear range of the sensor for xanthine as analyte were 6.54 µA mM^−1^, 0.01 µM, and 0.039–21 µM [94].

#### 2.2.6. Sulfite Dehydrogenase and Sulfite Oxidase

**Figure 13:**
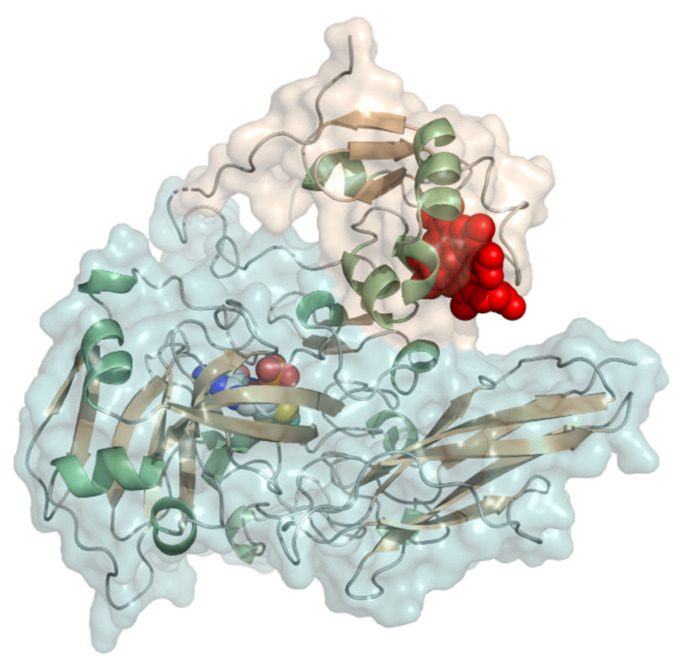
Crystal structure of *Gallus gallus* sulfite oxidase (PDB: 1SOX) with the Mo-domain colored in light cyan and the cytochrome domain in light red. The respective cofactors (Mo and heme) are shown as spheres.

Sulfite sensors are proposed to be useful, for instance, in the beverage industry. Sulfite dehydrogenase (EC 1.8.2.1; sulfite dehydrogenase (cytochrome)) is a molybdenum-containing enzyme with a size of around 40 kDa, existing as a heterodimer with an attached cytochrome *c*_551_ subunit (Figure 13). It is structurally distinct from sulfite oxidase. The dehydrogenase domain consists mostly of β-sheets. It catalyzed the oxidation of sulfite to sulfate. Direct electrochemistry with three midpoint potentials originating from the molybdenum or the heme of +177 mV, +211 mV, and −118 mV vs. NHE were determined.

Sulfite oxidase (EC 1.8.3.1) is a ~50 kDa homodimeric molybdohemoprotein with an isolated heme *b* domain being connected to the molybdopterin/iron/sulfur cluster domain by a polypeptide chain. An additional domain without any cofactor comprises flexible loops and beta-barrel like structures and structurally resembles proteins of the immunoglobulin family. The cytochrome domain structurally resembles *b*_5_-type cytochrome. O_2_ is used as co-substrate and substrates are reduced to sulphate under formation of H_2_O_2_. Sulfite oxidase for sulfite measurement was used as a recognition element on sensors as early as in the late 80s [95]. The DET of sulfite oxidase was demonstrated and a midpoint potential of −120 mV vs. Ag|AgCl was determined. The low midpoint potential allows amperometric measurements without interference of H_2_O_2_ [96,97]. Though DET was proposed for this enzyme, studies also employ mediators such as osmium polymers for a sulfite oxidase-based biosensor [98]. A 3rd generation biosensor was also tested with sulfite oxidase immobilized on indium tin oxide (ITO) electrodes with an obtained linear range from 2 to 20 µM sodium sulfate solution at 0 mV vs. Ag|AgCl. Currents between 20 to 245 nA (1–10 µM substrate) were reported [99].

#### 2.2.7. PQQ-Dependent Pyranose Dehydrogenase

**Figure 14:**
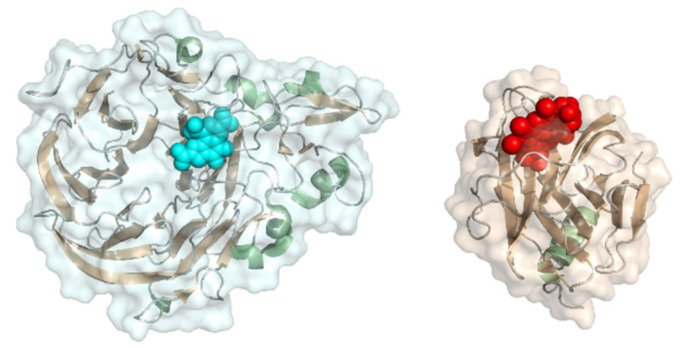
Crystal structure of *Coprinopsis cinerea* PQQ-dependent pyranose dehydrogenase with the dehydrogenase domain (PDB: 6JWF) colored in light cyan and the cytochrome domain (PDB: 6JT6) colored in light red. PQQ and heme b are shown as cyan and red spheres, respectively.

PQQ-dependent pyranose dehydrogenase (EC 1.1.2.B5, PQQ-dependent hemoquinoenzyme pyranose dehydrogenase) converts monosaccharides such as D-glucose and L-fucose. It consists of a CAZy AA12 PQQ-dependent dehydrogenase domain with a type 1 carbohydrate binding module (CBM_1) and a heme *b* cytochrome domain similar to that of *Phanerochaete chrysosporium* cellobiose dehydrogenase (Figure 14). The heme *b* cofactor mostly consists of β-sheets but has one helical structure on the protein surface (PDB: 6JT6). The size is ~90 kDa and the PQQ domain structure is dominated by a six-blade propeller fold. The PQQ-pyranose dehydrogenase must not be confused with the fungal FAD-pyranose dehydrogenase (Section 2.1.6) A detailed study showing substrate specificity on many other carbohydrates was published in 2020 [100]. Comparable to cellobiose dehydrogenase, PQQ-dependent pyranose dehydrogenase has separate dehydrogenase and cytochrome domains, connected by a functional linker. The heme *b* cofactor mostly consists of β-sheets but has one helical structure on the protein surface (PDB: 6JT6) and is structurally similar to the heme *b* domain of the *Neurospora crassa* cellobiose dehydrogenase. The DET of the *Coprinopsis cinerea* PQQ pyranose dehydrogenase was studied on carbon electrodes by Takeda et al. (2016) [101]. The group also characterized the isolated cytochrome *b* domain [102]. With cyclic voltammetry, they showed a midpoint potential of +130 mV vs. NHE at pH 7 for the full-length pyranose dehydrogenase on a carbon electrode modified with carbon nanoparticles.

#### 2.2.8. Bacterial PQQ-Dependent Type II Alcohol Dehydrogenase

**Figure 15:**
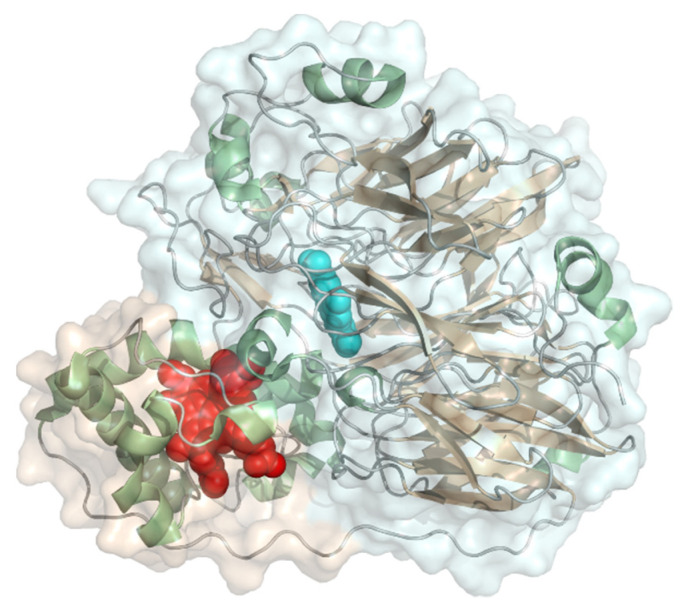
Crystal structure of *Pseudomonas putida* type II alcohol dehydrogenase (PDB: 1YIQ) comprising a PQQ-dependent dehydrogenase domain (light cyan) and a heme c-dependent cytochrome domain (light red). The respective cofactors are shown as colored spheres (PQQ, cyan; heme c, red).

Type II alcohol dehydrogenase (EC 1.1.9.1, quinohemoprotein ethanol dehydrogenase) is found mostly in *Pseudomonas* species and has a PQQ-carrying dehydrogenase domain and a cytochrome *c* domain connected via a flexible linker. Its molecular weight is 77 kDa and the PQQ domain structure consists of a six-stranded β-propeller fold, similar to other PQQ dehydrogenases (Figure 15). The exposed PQQ cofactor is presented towards the cytochrome domain. Because of its similar structure to cellobiose dehydrogenase, this enzyme is also recognized for application in biosensors. The natural electron acceptor is azurin. It oxidizes a wide range of primary and secondary alcohols but also aldehydes and sterols. Ubiquinone is used as co-substrate to produce acetaldehyde and ubiquinol. Use of this enzyme for bioelectrocatalysis was reported in 1993 by Ikeda and coworkers [103]. Surprisingly, there is no convincing publication for an applicable DET-based biosensor with this type of alcohol dehydrogenase.

Type III alcohol dehydrogenase (EC 1.1.5.5, membrane-associated quinohemoprotein alcohol dehydrogenase) is structurally distinct from type II alcohol dehydrogenases. It is membrane-attached with a PQQ-carrying subunit and a multiheme *c* subunit. Evidence for DET of this enzyme was reported in 1999 [104]. However, literature employing this type of alcohol dehydrogenase as recognition element in biosensors is proven and the reported biosensors are based on mediated electron transfer.

#### 2.2.9. Bacterial PQQ-Dependent Lactate Dehydrogenase 

This enzyme (EC 1.1.5.B3, quinone dependent L-lactate dehydrogenase) is a quinohemoenzyme with PQQ and heme *c* prosthetic groups. It has been expressed and purified out of a number of PQQ-dependent dehydrogenase encoding genes with unknown function from *Gluconobacter* sp., known for hosting a number of membrane-bound PQQ-dependent dehydrogenases like alcohol, aldehyde, glucose, and glycerol dehydrogenases. DET was demonstrated with cyclic voltammetry on carbon and gold electrodes [105]. The midpoint potential according to the cyclic voltammogram is close to +450 mV vs. Ag|AgCl, which is rather high. Cathodic peaks were small compared to clearly defined anodic peaks in a catalytic cyclic voltammogram after addition of lactate which the authors attributed to the large size and heme subunit orientation of the enzyme. Due to the big size of the protein, a large peak separation of almost 300 mV was also observed when the protein was adsorbed on carbon screen printed electrodes. A more detailed characterization of this enzyme is required to support DET.

### 2.3. Fusion Enzymes 

**Figure 16:**
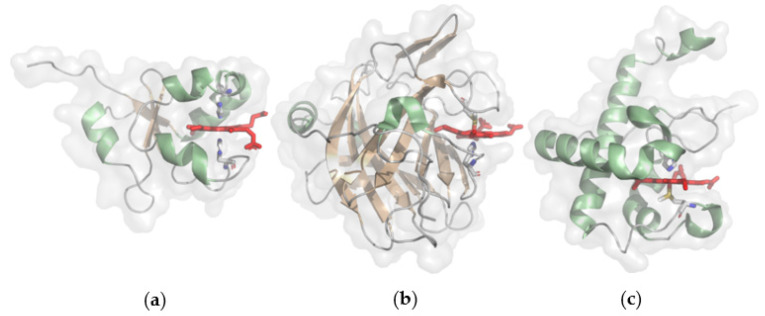
Cytochrome domain types with surface-exposed heme propionates (red sticks) frequently found in DET-capable multi-cofactor enzymes. (**a**) Heme *b* domain of *Gallus gallus* sulfitef oxidase (PDB: 1SOX) that structurally resembles *b_5_*-type cytochrome or *b*_2_ cytochrome domain of L-lactate dehydrogenase; (**b**) heme *b* domain of *Neurospora crassa* cellobiose dehydrogenases (PDB: 4QI7), which can be also found in PQQ-pyranose dehydrogenases; (**c**) heme *c*_551_ domain of *Pseudomonas putida* PQQ-dependent type II alcohol dehydrogenase (PDB: 1YIQ).

The presence of a cytochrome domain linked to the catalytic dehydrogenase domain as in cellobiose dehydrogenase, sulfite dehydrogenase, type II alcohol dehydrogenase as well as PQQ-dependent pyranose oxidase enables enzymes to perform DET. Fusing a cytochrome domain to enzymes that naturally do not have a heme domain aims to enable DET for these enzymes. Figure 16 presents cytochrome types usually found in naturally occurring multi-cofactor proteins. The cytochrome domain must have a suitable redox potential higher than the catalytically active domain. Cytochromes as direct electron transfer mediators were also reviewed in detail by Ma and Ludwig [106]. The *b*_5_/ *b*_2_-type cytochromes have usually low redox potentials of −50 to 0 mV vs. NHE, the cytochrome *b* domain of cellobiose dehydrogenase has a redox potential in a range of +50 to +150 mV vs. NHE depending on its origin and the pH. Cytochromes of the *c*-type have the highest redox potential in a rather broad range of +100 to +300 mV vs. NHE or even higher and occur mostly in PQQ enzymes. The *c*-type cytochrome domains in DET enzymes have a well exposed heme cofactor and are structurally slightly distinct to the typical mitochondrial cytochrome *c*, which have a more buried cofactor due to an extra peptide loop that covers the propionate groups. *b*-type hemes are inserted into the peptide via hydrophobic interactions, *c*-type hemes have basically the same structure as heme *b* but are covalently linked to the CXXCH motif of the peptide backbone. To achieve fast electron transfer and favorable diffusion properties, the cytochrome domain ideally is small and must fit sterically and electrostatically to the catalytic domain in addition to the requirement of a fitting redox potential.

#### 2.3.1. PQQ-Dependent Glucose Dehydrogenase Fused to a Cytochrome *c* Domain 

One of the very first publications where direct electron transfer of a fused protein was shown with amperometric techniques was published in 2004 [107]. The PQQ-glucose dehydrogenase of *Acinetobacter calocoaceticus* was fused to the cytochrome *c* domain of the two-domain ethanol dehydrogenase of *Comamonas testosteroni*. The fusion protein solution was mixed with graphite powder and paraffin to a carbon paste which was then applied to a carbon electrode. Amperometric measurements were performed at a high potential of approx. +300 mV vs. Ag|AgCl. A sensitivity of 0.13 µA mM^−1^ cm^−2^ was reported for the biosensor.

#### 2.3.2. FAD-Dependent Glucose Dehydrogenase Fused to a Minimal Cytochrome *c* Domain

The cytochrome *c* domain (magnetochrome) of magnetosome-associated protein MamP of *Magnetococcus marinus*, which in simple terms consists of the heme *c* binding motif CXXCH surrounded by a few amino acids, was fused to the FAD-dependent bacterial glucose dehydrogenase of *Burkholderia cepacia* [108]. Square wave voltammetry revealed a redox potential of approx. −90 mV vs. NHE attributed to the full-length magnetochrome and in agreement with values determined by spectroelectrochemical titration [109]. The fusion protein was trapped on the surface of a glassy carbon electrode with a dialysis membrane. The engineered protein was able to directly interact with the electrode, as shown from catalytic currents of more than ~2.5 µA cm^−2^ in the cyclic voltammogram [108].

#### 2.3.3. FAD-Dependent Glucose Dehydrogenase Fused to a Cytochrome *b* Domain

A glucose biosensor with the *Aspergillus flavus* glucose dehydrogenase fused to the *Phanerochaete chrysosporium* cellobiose dehydrogenase cytochrome domain was prepared [110]. Currents in the range of 2–4 nA were reported for this construct using multiwalled carbon nanotubes modified screen printed carbon electrodes. The measurements were performed at a relatively high voltage of approx. +400 mV vs. Ag|AgCl. The cytochrome domain of *Phanerochaete chrysosporium* cellobiose dehydrogenase has a redox potential of >200 mV vs. NHE [111]. Considering that free FAD itself already has a redox potential of around 400 mV vs. NHE, it cannot be excluded that the detected currents in this publication also include electron transfer mediated by free FAD. 

#### 2.3.4. FAD-Dependent Glucose Dehydrogenase Fused to Cytochrome *b*_562_

A fusion protein of the *Aspergillus flavus* glucose dehydrogenase and the cytochrome *b*_562_ of *Escherichia coli* was constructed [112]. For this fused construct, a gold electrode was modified with mesoporous carbon particles and the protein was crosslinked by glutaraldehyde. Measurements were performed at +400 mV vs. Ag|AgCl at 37 °C and showed an initial current of 100 nA and currents of approximately 150 nA to 600 nA upon addition of 0.1 to 75 mM glucose.

### 2.4. Performance of DET Enzymes 

Not all of the mentioned enzymes are equally suitable for 3rd generation biosensors. Heme containing enzymes like peroxidases are certainly promising catalysts for biosensors due to their high DET currents (Table 1). Peroxidases can be used for the detection of H_2_O_2_ or in bi-enzymatic setups together with H_2_O_2_ producing oxidases. In contrast, copper oxidases show good DET properties but usually have high redox potentials, which opposes the desired operation of 3rd generation biosensors at as low as possible potentials in order to avoid electroactive interferences. DET of FAD-dependent glucose oxidase and dehydrogenase is discussed controversially in the literature and was only shown with very specific electrode architectures. Consumption of O_2_ and formation of H_2_O_2_ is generally problematic when 3rd generation biosensors are operated at high potentials. 

The multi-cofactor enzymes presented here are equipped with an electron transfer unit capable of fast DET rates to electrodes. Low redox potentials of the usually involved heme domains in combination with high interdomain electron transfer rates make enzymes with a catalytic domain connected to an electron transfer domain very good candidates for application in 3rd generation biosensors. However, the availability and thus diversity of naturally occurring multi-cofactor enzymes is limited. In order to broaden the scope of 3rd generation biosensors for analytes of choice, rigorous protein engineering towards substrate specificity and selectivity of the available multi-cofactor enzymes is inevitable. An alternative strategy is the fusion of oxidoreductases to rationally selected electron transfer units like cytochromes. These fusion enzymes could widen the range for different analytes. Although currently reached DET rates are low, this approach is very likely to produce promising bioelectrocatalysts with a wide range of analytes for 3rd generation biosensors.

## 3. Electrode Materials and Modifications

The DET of an enzyme to an electrode surface is predetermined by the distance of the enzyme’s cofactor to the surface. The surface structure and properties of electrodes therefore greatly influence the DET efficiencies of the immobilized enzymes. Given a protein’s three-dimensional structure, the prosthetic group in most oxidoreductases is buried deeply within the protein shells. Thus, the direct electrochemical contacting of the prosthetic group by an electrode surface is rarely encountered [115,116,117]. In the past three decades, efforts have been made to design and engineer electrode surfaces for various enzymes to improve DET-based bioelectrocatalysis. The modified electrode surfaces generally favor an enzyme orientation that performs DET more efficiently than the bare electrode. The commonly used strategies may be assigned to one or more of the following three categories: (1) self-assembled monolayers (SAMs), (2) porous nanostructured electrode surfaces, and (3) nanomaterials modification electrodes. Additionally, modified electrode surfaces can also greatly improve enzyme immobilization and retain the stability of enzymes during catalysis. The primary aim of an electrode modification is to create an electron-transfer pathway that is as short as possible to connect the enzymes’ redox site and the electrode surface, because the interfacial electron transfer rate constant decreases exponentially with the distance [118,119,120].

### 3.1. Non-Modified Electrodes 

Despite the prevalence of electrode modification types of the above-named categories in the design of amperometric DET-based biosensors, several exceptions, that is, biosensors getting along without any electrode modification, have to be recognized. The first example is D-gluconate dehydrogenase, and it was reported to perform DET on bare glassy carbon, carbon paste, indium tin oxide (ITO) and gold electrodes without creating any mesoporous structure or modifying any nanomaterials or SAMs [121,122,123]. In these studies, the enzyme was generally adsorbed on the bare electrodes and was able to produce a pair of well-defined current peaks. The anodic current increased with addition of substrate D-gluconate. In the first publication, the catalytic current increased with D-gluconate up to a concentration of 5 mM on a carbon paste electrode. The mid-potential of the adsorbed D-gluconate dehydrogenase on ITO electrode was 0.05 V vs. Ag|AgCl and attributed to the heme *c* domain in the enzyme [123]. The DET of this enzyme was also investigated on commonly-used bare electrodes, such as Pt, Au, Ag and pyrolytic graphite electrodes [124]. Another typical example is the Type III alcohol dehydrogenase. It was also found to undergo DET on untreated carbon rods, graphite and screen-printed carbon electrodes [125]. The sensitivity of a screen-printed carbon electrodes-based ethanol biosensor, via the DET electrocatalysis of alcohol dehydrogenase, reached 179 µA cm^−2^ mM^−1^ [125]. Both alcohol dehydrogenase and D-gluconate dehydrogenase have three (or four) domains, which potentially allows for multiple electron transfer paths between enzymes and electrodes.

### 3.2. Electrodes Modified with Self-Assembled Monolayers

SAMs modification aims to create suitable surfaces for enzymes adsorption and further induce favorable orientations for DET between the enzymes and the electrodes. Meanwhile, various terminal groups with different surface charges and properties, mimicking the microenvironment in biological membranes, can improve the activity of immobilized enzymes. In this respect, the formation of a thiol-SAM on a gold electrode (or Au nanoparticles) undoubtedly is the most versatile and easiest method, because of the many commercially available thiols, the robust Au-S linkage and the simple preparation methods. Gold electrodes modified with a variety of thiol-SAMs have been widely used for DET enzymes, such as cellobiose dehydrogenase [44,126,127,128], fructose dehydrogenase [80,81,85], pyranose dehydrogenase [55,129], glucose dehydrogenase [78,130], peroxidases [34], sulfite oxidase [99], and others [118] (Table 2). Concerning the preparation and characterization of thiol-SAMs modification, Yan et al. reviewed the frequently used alkanethiol- and thiophenol-SAMs with alkyl-, amino-, hydroxyl- or carboxyl-terminal groups for heme-containing enzymes, blue copper oxidases, and FeS-cluster hydrogenases and concluded that the strategic selection of SAM in accordance with the enzyme properties determines the electrocatalysis performance of DET enzymes [118]. In the review about methodologies for “wiring” redox enzymes to electrodes by Yates et al., some strategies of thiol SAMs selection for the specific enzymes were covered. For example, amino-terminated SAMs generally favor enzymes bearing negative surface charges but they also emphasized the complexity in optimizing such a thiol SAM-enzyme combination [131]. 

Thiol-SAMs have been frequently applied for cellobiose dehydrogenases in both enzymatic kinetics investigation and 3^rd^ generation glucose or lactose biosensors. Lindgren et al. demonstrated the immobilization of *Phanerochaete chysoporium* cellobiose dehydrogenase on a cysteamine-modified gold disk electrode, the catalytic current for cellobiose substrate was about 4.55 μA mM^–1^ cm^–2^ at an optimized pH 3.50 [126]. Stoica et al. systematically compared the DET ability of cellobiose dehydrogenases on 10 different alkanethiol-modified gold electrodes. For example, the cyclic voltammogram of *Trametes villosa* cellobiose dehydrogenase on 4,4′-aldrithiol showed a current over 350 nA upon oxidation of 1 mM cellobiose at pH 3.5. Considering the biocatalytic currents of cellobiose oxidation, it was found that thiols with OH- terminal groups are the best options for orientation of the cellobiose dehydrogenases [132]. Glucose is one of the natural substrates of many cellobiose dehydrogenases. For example, *Crassicarpon thermophilum* cellobiose dehydrogenase has a remarkably high turnover numbers for oxidation of glucose. Antiochia et al. reported the DET of this cellobiose dehydrogenase for glucose oxidation reaction on gold nanoparticles modified with a mixture SAMs of 4-aminothiophenol and 4-mercaptobenzoic acid [74]. This 3rd generation glucose biosensor exhibited an extended linear range from 0.02 to 30 mM with a sensitivity of 3.1 µA cm^−2^ mM^−1^. The linear detection range is much broader than most of the first-generation glucose biosensors. 

Three thiol-SAMs with different terminal groups (4-mercaptobenzoic acid, 4-mercaptophenol, and 4-aminothiophenol) were applied for fructose dehydrogenase, it was found that 4-mercaptophenol-modified porous gold electrode generated a well-pronounced catalytic current and exhibited the best analytical performance, a detection limit of 0.3 μM fructose, a linear range of 0.05–5 mM, and a sensitivity of 175 μA cm^–2^ mM^–1^ [85]. However, the contribution of the porous structure in their gold electrode to the catalytic current has not been discussed or compared with a plane gold electrode. By comparison of the relevant studies, it seems the SAM with a hydroxyl terminal group has a four-fold higher electrocatalytic current than the SAM with an alkyl group [133,134] (Table 2). This indicates that the hydrophobic terminal group is less suitable for fructose dehydrogenase to generate high DET currents. In addition to the two enzymes, the thiol-SAMs (dithiobis(succinimigyle hexanoate), dithiobis(succinimidyl octanoate), and dithiobis(succinimidyl undecanoate)) with different molecular length have been used for the detection of the DET of FAD-dependent glucose dehydrogenase. The electrocatalytic current was found to decrease with the increase of the SAM length [130]. As to tobacco peroxidase, Gaspar et al. reported that the positively charged SAM (hexamethylcystamine) generated significantly higher electrocatalytic current (17.9 µA M^−1^) than the negatively charged SAM 3-carboxy-propyldisulfide (2.5 µA M^−1^) [34]. Very recently, a 2-mercaptoethanol SAM-coated gold electrode was found to greatly improve DET electrocatalysis for the PQQ-dependent pyranose dehydrogenase, resulting in a 10-times higher current density than the bare electrode with the same enzyme loading [55]. Notably, the DET of glucose oxidase to a thiol-modified gold electrode was obtained by immobilizing the enzyme on a self-assembled monolayer of 3,3′-dithiobis-sulfocinnimidylpropionate (DTSSP) [135].

**Table 2:** Various SAMs applied for DET-based biosensors.

SAM Forming Molecule	SAM Terminal Group	Electrode Material	Enzyme	Analyte	Current (Density) or Sensitivity	Ref.
Cysteamine	amino	Au	Cellobiosedehydrogenase	1 mM cellobiose	est. 4.55 μA cm^–2^	[126]
Cysteamine	amino	Au-NPs	Glucose dehydrogenase	glucose	715 µA mM^−1^ cm^−2^	[78]
4,4′-Aldrithiol	hydroxyl	Au	Cellobiose dehydrogenase	1 mM cellobiose	est. 0.35 μA	[132]
11-Mercapto-1-undecanol	hydroxyl	Au	Cellobiose dehydrogenase	10 mM lactose	est. 35 µA cm^−2^	[127]
4-Mercaptobenzoic acid	carboxyl	Au	Cellobiose dehydrogenase	10 mM lactose	est. 20 µA cm^−2^	[127]
4-Mercaptophenol	hydroxyl	Au	Cellobiose dehydrogenase	10 mM lactose	est. 6 µA cm^−2^	[127]
2-Mercaptoethane	alkyl	Au	Fructose dehydrogenase	200 mM fructose	est. 40 µA cm^−2^	[134]
2-Mercaptoethanol	hydroxyl	Au	Fructosed ehydrogenase	100 mM fructose	est. 150 µA cm^−2^	[133]
4-Mercaptophenol	hydroxyl	Au	Fructose dehydrogenase	fructose	175 μA mM^–1^ cm^–2^	[85]
Dithiobis(succinimidyl hexanoate)	succinimidylester	Au	FAD-Glucose dehydrogenase	5 mM glucose	est. 2.8 µA cm^−2^	[130]
4-Aminothiophenol/ 4-Mercaptobenzoic acid	hydroxyl/ carboxyl	Au	Cellobiose dehydrogenase	glucose	3.1 µA mM^−1^ cm^−2^	[74]
Dithiobis-(succinimidyl octanoate)	succinimidyl ester	Au	FAD-Glucose dehydrogenase	5 mM glucose	est. 1.2 µA cm^−2^	[130]
Hexamethylcystamine	amino	Au	Peroxidase	H_2_O_2_	0.018 µA mM^−1^	[34]
3-Carboxy-propyldisulfide	carboxyl	Au	Peroxidase	H_2_O_2_	0.0025 µA mM^−1^	[34]
1,4-Phenylenediamine	amino	SWCNT	Cellobiose dehydrogenase	5 mM lactose	500 μA cm^−2^	[114]

est: this value was extracted or calculated from data given in the reference, e.g., from a cyclic voltammogram.

Non-thiol SAMs, aryl amine and aryl diazonium are generally used for carbon materials surface modification. For example, *p*-aminobenzoic acid or *p*-phenylenediamine can modify CNTs with a negatively and positively charged surface, respectively. DET electrocatalysis of cellobiose dehydrogenase on the *p*-aminophenyl SAM (450 μA cm^−2^) produced roughly four times higher currents than *p*-aminobenzoic acid (110 μA cm^−2^) at 200 mV vs. NHE in a 5 mM lactose solution buffered at pH 3.5 [114]. This result is in accordance with the previously mentioned thiol-SAMs; therefore, the positive terminal SAMs are in favor of DET for cellobiose dehydrogenase regardless of electrode materials.

#### 3.2.1. Mesoporous or Nanostructured Electrodes

Nanoscale porosity is classified by IUPAC according to the pore size into microporous (≤2 nm), mesoporous (2–50 nm), and macroporous (≥50 nm) materials. Mesoporous structures are most commonly encountered in electrodes and are either an inherent property or created by a specific treatment, such as surface etching techniques. Concerning the electron transfer theory of adsorbed enzymes in mesoporous structures, a model was established by Adachi and coworkers [136]. They hypothesized that the curvature effect of the mesopores increases the probability of an enzyme’s orientation suitable for DET and also performed simulations with three-dimensional models of randomly orientated enzymes in mesopores. The results showed that when the radius of a pore is equal to the radius of the enzyme, the DET current could reach a maximum. However, enzyme loading in mesopores structures decreases with the size of pores and the adsorption of enzymes is strongly hindered when the pores possess similar sizes as the enzymes. The effective loading of enzymes requires pore sizes three to four times larger than the enzymes’ sizes. Therefore, the sizes of mesopores on electrodes should be balanced for both the curvature effect and the maximum of enzyme loading. The application of mesopores and nanostructured electrodes to improve DET of cellobiose dehydrogenase for biosensors and enzymatic fuel cells can be found in Bollela et al. [137]. Here, we will focus on the studies where the mesoporous and nanostructured graphite or gold electrode enabled the DET of various enzymes.

#### 3.2.2. Mesoporous Graphite Electrodes

The high porosity of spectroscopic graphite can increase the specific surface area and enhance the adsorption of enzymes. Blanford reported the Brunauer–Emmett–Teller surface area of a commonly polished graphite electrode can be approximately 10^4^ times greater than its geometric area [138]. They proposed that the enzyme loading on this porous graphite is potentially one to two orders of magnitude higher than on a planar one. The electron transfer characteristic of cellobiose dehydrogenase on the porous graphite was first investigated by Larsson et al. in 1996 [71]. They found the physically adsorbed cellobiose dehydrogenase gave a much higher (over 10-fold) electrocatalytic current than the one co-immobilized with Os-polymers on graphite electrodes. It was proposed that the intrinsic heme *b* domain within cellobiose dehydrogenase surpassed the external electron mediator in transferring electrons between the FAD and the electrode. In the follow-up studies, they presented more solid evidence to claim the DET of cellobiose dehydrogenase on the graphite electrode [126], and reported a *Phanerochaete sordida* cellobiose dehydrogenase-based 3rd generation lactose biosensor with a sensitivity of 1100 µA mM^−1^ cm^−2^ and a linear range from 1 to 100 µM [73].

Basal-plane pyrolytic graphite electrodes were utilized to study fructose dehydrogenase [139]. The catalytic currents obtained from oxidation of 100 mM fructose by the adsorbed enzyme was ∼9.0 µA cm^−2^ in phosphate buffer (pH 5.0) without redox mediator, but decreased to almost zero in pH 7.0 [139]. DET between the deglycosylated FAD-dependent pyranose dehydrogenase and porous graphite was discovered in another study, but the catalytic current from oxidation of glucose was not sufficiently high for an efficient biosensor [46]. 

#### 3.2.3. Mesoporous Gold Electrodes 

Nanostructured gold is a well-studied electrode material for enzymes to establish DET. Mesoporous gold electrodes can be prepared by the anodization of gold in the presence of oxalic acid or glucose as a reductant [140]. This electrochemical fabrication method is easy to conduct and allows an easier control of pore and channel sizes. The porous gold electrode enabled the DET of *Aspergillus terreus* glucose dehydrogenase, which hardly generated electrocatalysis on common counterpart-electrodes. The cyclic voltammogram of *At* glucose dehydrogenase-modified porous gold electrode showed a pair of current peaks at ca. −0.1 V vs. Ag|AgCl under non-turnover conditions and the anodic current raised to 0.1 mA cm^−2^ in the presence of 100 mM glucose [43]. Bollella et al. applied highly porous gold for immobilization of fructose dehydrogenase, reaching electrocatalytic currents for oxidation of 10 mM fructose of ca. 120 µA cm^−2^, and even higher currents when modifying the gold pores with a thiol-SAM [85]. Mesoporous or nanostructured gold surfaces were also used for enabling or promoting the DET of peroxidase [140], bilirubin oxidase [141], and laccase [142]. 

### 3.3. Electrodes Modified with Nanomaterials

**Figure 17:**
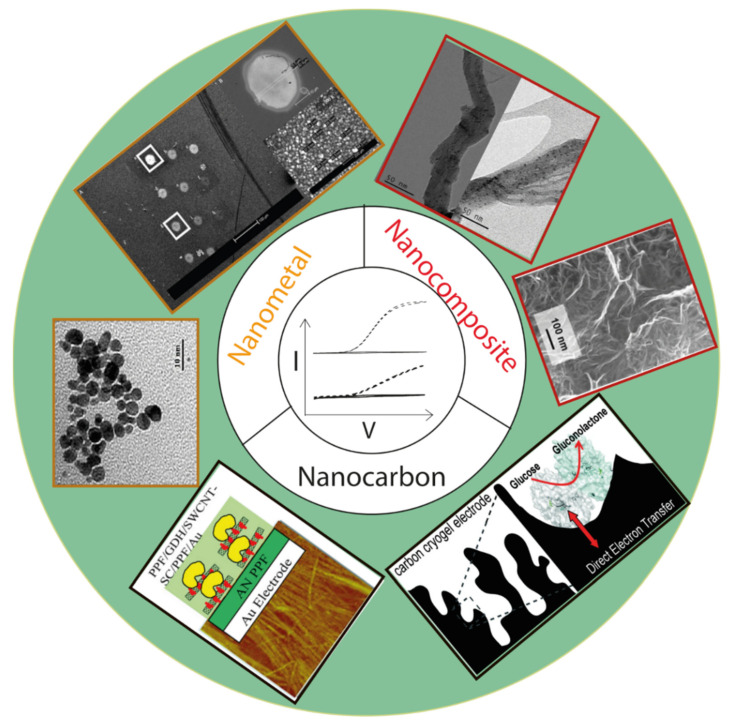
Overview of the examples of commonly employed nanomaterials for enabling or enhancing DET electrocatalysis. Nanocarbon: carbon cryogel for PQQ-glucose dehydrogenase [50], SWCNT for FAD-glucose dehydrogenase [144]; nanometal: polyethyleneimine-stabilized Au nanoparticles for cellobiose dehydrogenase [153], mixed thiol-SAMs modified Au nanoparticles for cellobiose dehydrogenase [127]; nanocomposites: PtNPs-MWCNT and PdNPs-MWCNT for cellobiose dehydrogenase [154], CNT-supported reduced graphene oxide for horseradish peroxidase [151].

It is widely reported that nanomaterials can improve the DET of various enzymes, or even establish DET for some enzymes that otherwise failed on plane electrode surfaces. The most often studied nanomaterials include single-walled or multi-walled carbon nanotubes, metal nanoparticles, graphene, and nanocomposites (e.g., carbon nanotube-Pt nanocluster), etc. (Figure 17). A comprehensive theoretical basis for the effect of nanomaterial size and surface properties on DET is still lacking and beyond the scope of this review. We report studies demonstrating an established/enhanced DET of enzymes by nanomaterials and the performance of the built biosensors.

In this respect, glucose oxidase is an interesting topic. Since the first publication in 1982 [143], a number of studies reported that glucose oxidase exhibits DET at nanomaterials modified electrodes. However, as mentioned earlier, only a small number of articles presented some evidence for DET between glucose oxidase and their modified electrodes [41]. Two of them reporting DET to carbon nanomaterial modified electrodes or hybrids with metal nanomaterials will be covered in the following two sections.

#### 3.3.1. Carbon Nanomaterials 

The most commonly employed nanomaterials that promote/establish DET are given in Table 3. Carbon nanotubes (CNTs) promoted the direct electrocatalysis of glucose for both FAD-glucose dehydrogenase (110 μA cm^−2^ mM^−1^) [144] and PQQ-glucose dehydrogenase (500 μA cm^−2^ at 5 mM glucose) [51]. The high catalytic current from glucose oxidation in these CNTs surface is unambiguous and has the great promise of biosensor applications. Other carbon nanoparticles such as Ketjen Black [145] and carbon cryogel [50] also have a large specific surface area, a graphite-rich composition, and a high conductivity, which improves enzyme immobilization and possibly the orientation of the enzyme with respect to the electrode [146]. PQQ-glucose dehydrogenase immobilized in a carbon cryogel with a pore size of about 20 nm generated 930 μA cm^−2^ catalytic current in the presence of 300 mM glucose [50]. This catalytic current equals another study where 1000 μA cm^−2^ was achieved using FAD-glucose dehydrogenase and Pt nanoclusters in presence of 100 mM glucose [43]. However, FAD-glucose dehydrogenase on single layer graphene [147] generated a three to four orders of magnitude lower catalytic current (0.3 μA cm^−2^) compared with both, the carbon nanoparticles and the CNTs. This implies that the zero-dimensional and one-dimensional nanomaterials are better at promoting the DET of FAD-glucose dehydrogenase than two-dimensional graphene. The carbon nanoparticles with three dimensions at nanometer sizes could maximize the optimal orientation effect and therefore present the best DET performance. On the other hand, a heme *b* domain-fused FAD-glucose dehydrogenase was also immobilized on SWCNT, but generated quite low catalytic current, 0.011 μA (ca. 0.15 μA cm^−2^, recalculated by the authors) for oxidation of 5 mM glucose [148]. This value is roughly three orders of magnitude lower than the native FAD-glucose dehydrogenase on SWCNT (110 μA cm^−2^ for 1 mM glucose). This might imply that native FAD-glucose dehydrogenase’s ability to perform DET-type electrocatalysis on CNT surface is greater than the heme *b* domain fused mutant.

An aqueous dispersion of reduced graphene oxide, multi-walled carbon nanotubes, and glucose oxidase on a glassy carbon electrode enabled the direct electron transfer of glucose oxidase to the electrode material with currents as high as 72 µA µg^−1^ enzyme [149]. This is one of the very few works of carefully addressed direct electrochemistry of glucose oxidase.

Carbon nanofibers [150], Ketjen Black [27], and CNT-supported reduced graphene oxide [151] were all used to study the DET of horseradish peroxidase, and the electrocatalytic responses for reducing H_2_O_2_ were 0.6 μA mM^−1^ cm^−2^, 4800, μA mM^−1^ cm^−2^, and ca. 5000 μA mM^−1^, respectively. Given that many carbon nanomaterials can also catalyze the reduction of H_2_O_2_ at any electrodes, it is important to discriminate the bio-electrocatalysis of horseradish peroxidase from the electrocatalysis of nanomaterials. In most cases, the synergy between the nanomaterials and the enzyme contributed to even higher catalytic currents, but the sole contribution of horseradish peroxidase was not carefully examined.

Carbon nano-fibers and -particles were also employed for the DET of fructose dehydrogenase [82,152]. When the enzyme was immobilized on hollow carbon nanoparticles, it generated remarkably high current response in the presence of 200 mM fructose (Table 3). In the other study, carbon nanofibers with a low oxygen/carbon ratio (ca. 5%) generated the highest catalytic current, 1 mA cm^−2^, and half of the optimum current was obtained on the nanofibers with a 30% oxygen/carbon ratio. Presumably the oxygen-containing functional groups affect the orientation of fructose dehydrogenase or the rate of electron transfer at the interface between the carbon nanomaterials and the enzymes [82].

**Table 3:** Nanomaterials, modifications, and performance of DET enzymes.

Nanomaterials	Material Properties	Comment	Enzyme	Analyte	Current (Density) Sensitivity	Ref.
Ketjen Black	large surface areas	miniaturized electrode	FAD-glucose dehydrogenase	5 mM glucose	est. 174 μA cm^−2^	[145]
Ketjen Black	mesoporous		horseradish peroxidase	H_2_O_2_	4.8 mA mM^–1^ cm^–2^	[27]
carbon cryogel	pore size of 20 nm		PQQ-glucose dehydrogenase	300 mM glucose	930 μA cm^−2^	[50]
carbon particles	hollow structure, particle size ~40 nm		Fructose dehydrogenase	200 mM fructose	est. 12 mA	[152]
SWCNT		heme *b* domain fused	FAD-glucose dehydrogenase	5 mM glucose	est. 0.011 μA	[148]
SWCNT		Poly(methoxyaniline sulfonic Acid) modified Au-electrode	PQQ-glucose dehydrogenase	5 mM glucose	500 μA cm^−2^	[51]
SWCNT	sodium cholate assisted dispersion	acetonitrile plasma-polymerized film (2 nm) on Au-electrode	FAD-glucose dehydrogenase	glucose	110 μA cm^−2^ mM^−1^	[144]
SWCNT	oxidatively shortened CNT		cellobiose dehydrogenase	glucose	0.22 μA cm^−2^ mM^−1^	[155]
graphene	single layer	covalent immobilization of glucose dehydrogenase	FAD-glucose dehydrogenase	20 mM glucose	est. 0.3 μA cm^−2^	[147]
graphene/ SWCNT	highly conductive, no stabilizers	final polyethyleneimine layer	FAD-glucose oxidase	glucose	72 μA μg^−1^	[149]
Pt- nanoclusters		porous gold electrodes	FAD-glucose dehydrogenase	100 mM glucose	est. 1000 μA cm^−2^	[43]
Pt and Pd NPs	particle sizes Pt 1.3– 2.5 nm, Pd 2.5–5 nm	Nanohybrids with CNT	cellobiose dehydrogenase	lactose	Pt: 3.07 μA mM^−1^ Pd: 3.28 μA mM^−1^	[154]
carbon nanofibers	cup-stacked structure controlled O/C ratio		fructose dehydrogenase	200 mM fructose	est. 1 mA cm^−^^2^	[82]
carbon nanofibers	edge-plane surface, cup-stacked		horseradish peroxidase	H_2_O_2_	est. 0.6 μA cm^−2^ mM^−1^	[150]
Au-NPs		4-aminothiophenol modification	alcohol dehydrogenase	50 mM glycerol	510 μA cm^−2^ mM^−1^	[156]
Au-NPs		3D printed graphene/polylactic electrode	horseradish peroxidase	H_2_O_2_	est. 65 μA mM^−1^	[31]
Au-NPs/graphene/PEI	hybrid	glutaraldehyde crosslinking	FAD-glucose oxidase	glucose	93 μA mM^−1^ cm^−2^	[157]

est: this value was extracted or calculated from data given in the reference, e.g., from a cyclic voltammogram.

#### 3.3.2. Metal Nanomaterials

Au nanoparticles modified with 4-aminothiophenol were used with alcohol dehydrogenase as bioanodes in biofuel cells [156]. The Au nanoparticles endow the bioanode with effective catalytic DET currents of 510 and 280 µA cm^–2^ upon oxidation of 50 mM glycerol and glyceraldehyde, respectively. The results suggest great promise in the fabrication of 3rd generation glycerol biosensor using this modified electrode. The 4-aminothiophenol SAM on the nanoparticles might play an important role in the establishment of DET. Au-NP was also applied to enhance the DET of horseradish peroxidase. Marzo et al. integrated Au-NPs onto 3D printed graphene/polylactic electrode, and the H_2_O_2_ biosensor had a sensitivity of 65 μA mM^−1^. They also systematically investigated the different electrocatalytic current from gold nanoparticles and horseradish peroxidase (Table 3) [31]. Pt/Pd nanoclusters [154] have been used for the immobilization of cellobiose dehydrogenase for DET-based glucose or lactose biosensors. Due to the interdomain electron transfer pathway of cellobiose dehydrogenase, the main function of the metal nanomaterials in these studies is to enhance the electrocatalytic current of DET, which can actually occur on bare graphite electrodes. For example, the electrocatalytic currents of the lactose biosensor based on Pt/SWCNT-modified electrodes is about 20% higher than those on sole SWCNT modified electrode (2.60 μA mM^−1^) and 200% higher than those on bare electrode (0.92 μA mM^−1^) [154].

A hybrid comprising metal and carbon nanomaterials for the fabrication of a glucose oxidase-based glucose biosensor was presented by Rafighi et al. [157]. They immobilized a mixture of graphene, Au nanoparticles, and polyethyleneimine on a gold electrode and crosslinked the enzyme via glutaraldehyde. The result was a glucose biosensor with a sensitivity of 93 µA mM^−1^ cm^−2^, a linear range between 1 and 100 µM, and an LOD of 0.32 µM. This work also numbers among the few sound DET studies employing glucose oxidase.

### 3.4. Performance of Electrode Materials and Modifications

A great number of thiol-based SAMs applied in 3rd generation biosensors are given in literature and only a selection of them is presented here. Comparison of data to find the most suitable thiol-properties for individual enzymes is often difficult because of missing information. Nevertheless, it can be stated that there is no universal thiol guaranteeing good results for enzyme immobilization and electrochemical communication. Specific enzymes prefer specific terminal groups on surface modifications. *Neurospora crassa* cellobiose dehydrogenase was tested with thiols of varying type and terminal groups. Out of short- and long-branched as well as phenolic thiols with hydroxyl-, carboxyl-, and amine terminal groups, 4-mercaptobenzoic acid worked best with *Neurospora crassa* cellobiose dehydrogenase and resulted in the highest catalytic current [158], whereas *Phanerochaete sordida* cellobiose dehydrogenase immobilized on SWCNT gave higher currents with positive amino groups over carboxy groups [114]. 

In contrast to thiol-based self-assembled monolayers, which aim to protect the enzyme from denaturation on noble metal electrodes while also providing direct electrochemical communication, the use of porous electrodes and nanomaterials of any kind aims towards the enlargement of the specific electrode area available for direct electrochemical contact with the enzyme. The combination of nanomaterials and electrode modifications in order to achieve high current outputs is ongoing and new materials and modifications are tested and published. These results (enzyme binding and DET current) are almost always enzyme-specific. The elucidation of an enzyme’s DET rate in regard to electrode surface properties needs defined conditions, e.g., a true monolayer of the enzyme immobilized in a defined orientation. Such studies are based on a combination of protein engineering and specific coupling of, e.g., surface-exposed cysteines via maleimide to defined electrode surfaces [159].

## 4. Conclusions and Future Perspectives

Direct electrochemistry of oxidoreductases is utilized to generate biosensors for simple and low-priced on-line quantification of analytes. The analyte spectrum covers a broad range of relevant analytes like saccharides, phenolic compounds, and small molecules such as sulfite or alcohols, which are relevant for environmental monitoring, compounds of the food industry, and products of biotechnological processes. Many reports in the literature focus on glucose detection because of the high medical relevance for blood glucose determination, although much more enzymes with suitable electrochemical properties exist and can be applied to detect other substrates. Several of those are capable of DET and can be used for 3rd generation sensors when combined with the correct combination of electrode materials and various electrode modifications. The performance of DET-based biosensors has to be carefully reported and the necessary control experiments to rule out mediated electron transfer should be provided. Despite this point of caution, many studies on DET enzymes and 3rd generation biosensors are performed with great care and show a high level of insight and expertise. Some research groups particularly focus on the optimized electrode material design employing nanomaterials and investigate optimized fabrication and protein immobilization strategies to achieve biosensors with higher sensitivity and stability. To date only one commercially available DET-based amperometric 3rd generation biosensor (LactoSens) exists compared to a great number of existing 1st and 2nd generation biosensors. Since not all oxidoreductases are capable of DET, the fusion of enzymes with electron-transferring cytochromes is a very promising strategy to increase the number of bioelectrocatalysts for 3rd generation biosensors and to increase the number of analytes.

Author Contribution: F.S. and H.C. prepared the first draft of the manuscript and compiled the tables and drew figures together with S.S. S.S. and R.L. critically reviewed the data and wrote the final draft of the manuscript.

## Figures and Tables

**Table 1:** Various analytes detected by DET-capable enzymes in 3rd generation biosensors.

Analyte	Prosthetic Group	Enzyme	Electrode Base	Electrode Modification	Sensitivity [µA mM^−1^ cm^−2^]	Ref.
Theophylline	heme	Cytochrome P450	SPGE	+/− DDAB *	0.05	[39]
Theophylline	heme	Cytochrome P450	Au		52.1	
H_2_O_2_	heme	Horseradish peroxidase	Au		1400–1500	[113]
H_2_O_2_	heme	Soybean peroxidase	GC	SWCNT	16.625 µA mM^−1^	[33]
H_2_O_2_	heme	Tobacco peroxidase	Au	SAM	ND	[34]
PhenolsDiamines	heme	Tobacco peroxidase	Au, SPGE	SAM	ND	[35]
L-Fucose	PQQ	Pyranose dehydrogenase	Au	Au-NPs	3.12	[54]
Lactose	FAD, heme *b*	Cellobiose dehydrogenase	GC	SWCNT/aryldiazonium	500 µA cm^−2^	[114]
Fructose, food sample	FAD, 3 heme *c*	Fructose dehydrogenase	Au	porous Au	175	[84]
L-Lactate (saliva, sweat)	FMN, heme *b*	Lactate dehydrogenase	Au	Au-NPs	ND	[92]
Glucose	PQQ, heme *c*	Glucose dehydrogenase/cytochrome *c*	graphite/paraffin paste	/	131.8	[107]
Glucose	FAD, heme *c*	Glucose dehydrogenase/cytochrome *c*	GC	/	ND	[108]
Glucose	FAD, heme *b*	Glucose dehydrogenase, cytochrome *b*	SPGE	MWCNT	ND	[110]
Glucose	FAD, heme *b*	Glucose dehydrogenase, cytochrome *b*_562_	Au	mesoporous carbon particles	2.14–8.57 µA cm^−2^	

* DDAB, didodecyldimethylammonium bromide.
